# Increased risk of SARS-CoV-2 infection and COVID-19 death among older patients at long-term care hospitals in Korea

**DOI:** 10.3389/fpubh.2023.1235745

**Published:** 2023-07-25

**Authors:** Jeong-Yeon Seon, Sunjea Kim, Min Kyoung Lim, In-Hwan Oh

**Affiliations:** ^1^Health Insurance Research Institute, National Health Insurance Service, Wonju, Republic of Korea; ^2^Department of Preventive Medicine, Kyung Hee University School of Medicine, Seoul, Republic of Korea

**Keywords:** older patients, long-term care hospital, COVID-19, SARS-CoV-2, infection, mortality

## Abstract

**Introduction:**

Long-term care hospitals are known to be vulnerable to SARS-CoV-2 infection and death given their numerous older chronic disease patients. However, the actual effect of long-term care hospital admission is not well known in Korea; hence, this study sought to analyze the effect of long-term care hospitalization on SARS-CoV-2 infection and COVID-19 death by correcting for patients’ characteristics.

**Methods:**

This cross-sectional study used the data from K-COV-N cohort, which is linked to the National Health Insurance Service and the Korea Disease Control and Prevention Agency; it analyzed 70,373 individuals aged ≥60 years, who had been tested for COVID-19 between January 1 and May 30, 2020 (KDCA-NHIS-2020-1-601). Patients admitted to a long-term care hospital were defined as those with a confirmed history of hospitalization within 30 days of the COVID-19 testing date. The final data analysis was performed in December 2022. Logistic regression analysis of the national data was employed to determine the association between long-term care hospital admission, the risk of SARS-CoV-2 infection, and death from COVID-19. The odds ratios for SARS-CoV-2 infection and death from COVID-19 were calculated by adjusting for sex, age, residential area, health insurance premium, disability, and the Charlson Comorbidity Index.

**Results:**

Older patients at long-term care hospitals had a high risk of SARS-CoV-2 infection (OR:2.91, 95% CI:2.33–3.64) and death from COVID-19 (OR:3.58, 95% CI:2.13–6.02). A difference in SARS-CoV-2 infection risk was observed based on residential area, health insurance premium (economic level), and disability; no difference was observed for COVID-19 mortality risk.

**Discussion:**

Admission to a long-term care hospital itself could be a risk factor for SARS-CoV-2 infection and the consequent high mortality risk after adjusting for sex, age, disability, and comorbidities. Patients are at high risk of infection through contact with workers, leading to death; therefore, quarantine policies for workers must be strengthened.

## 1. Introduction

The coronavirus disease 2019 (COVID-19) first occurred in Wuhan, China, in December 2019; it spread worldwide and continued to do so for a long period of time. By 2022, the cumulative number of COVID-19 cases confirmed in Korea was 29,059,273, accounting for 56.3% of the total population; the cumulative number of deaths was 32,156, with a fatality rate of 0.11% (1). In Korea, COVID-19 vaccination began in February 2021, prioritizing workers at high-risk medical institutions and residents of collective facilities (2). Countries which spearheaded COVID-19 vaccination, such as the United States and the United Kingdom, also recommended vaccinating these people first, viewing long-term care facility residents and workers as groups vulnerable to SARS-CoV-2 infection (3, 4).

Long-term care hospitals in Korea are medical institutions that provide medical care to patients who need long-term hospitalization, and mainly offer rehabilitation and nursing services rather than surgery (5). As of 2020, older patients accounted for 80.3% of all people admitted to long-term care hospitals, while the average annual hospitalization period was 157 days. Long-term care hospitals and facilities have similar characteristics (6). Many previous studies have analyzed the risk of SARS-CoV-2 infection or death in long-term care facility residents (7–9); however, studies which quantitatively reveal that hospitalization in long-term care hospitals is a risk factor for COVID-19 are insufficient. It is difficult to determine the effect of hospitalization on COVID-19 because of the characteristics of patients admitted to long-term care hospitals, i.e., they are older, chronically ill, and undergoing rehabilitation (6). In the current study, the risk of SARS-CoV-2 infection and COVID-19 death in older patients admitted to long-term care hospitals was analyzed by controlling for the spread of COVID-19 and other factors that might affect clinical results. Based on this, we intend to support the prioritization of COVID-19 quarantine policies.

## 2. Methods

### 2.1. Data collection and study participants

**Figure 1:**
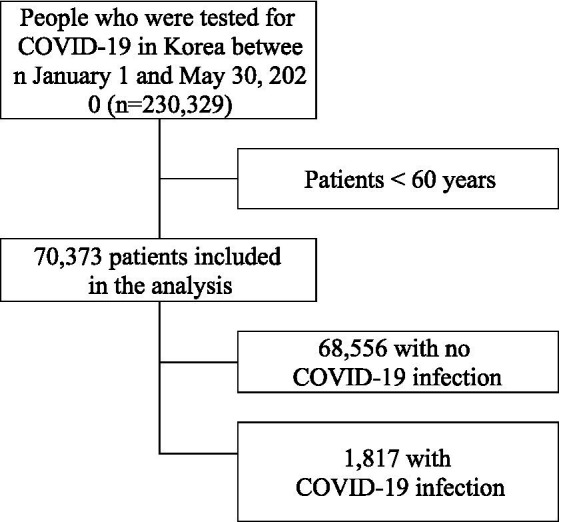
Flow chart for patients included in the analysis.

In this study, we used the data from K-COV-N cohort(Korea Disease Control and Prevention Agency-COVID19-National Health Insurance Service cohort) - which is linked Korea Disease Control and Prevention Agency (KDCA) and the National Health Insurance Service(NHIS) - to analyze the association between SARS-CoV-2 infection and COVID-19 death and older inpatients at long-term care hospital. The research number of this study is KDCA-NHIS-2020-1-601. Older individuals aged 60 or over were targeted using the K-COV-N cohort, which includes national qualifications, insurance premiums, health checkup results, and treatment details of COVID-19 patients and controls. The NHIS is the only insurer in the Korean healthcare system, and all medical issues, including those requiring long-term care hospitals, are included in the health insurance data (10). In this study, analysis focused on 70,373 people aged 60 or older from a pool of 230,329 people who took the COVID-19 test between January 1, 2020 and May 30, 2020. Korea’s payment system of healthcare is based on fee-for-service, and there is the Korean medical practice code that is Electronic Data Interchange code for each medical service. Those who got tested for COVID-19 were defined as those for whom a PCR test codes (D658900, D658800, D658404, D658498, D658499, D658298, D680001, and D662000) were claimed. In the early stages of COVID-19, the subjects of diagnostic tests were frequently revised and gradually expanded (Supplementary Data 1). Subjects for COVID-19 diagnostic tests included those who developed symptoms or had an epidemiological link and were diagnosed through PCR testing; the cost of the test was borne by the government (11). Of those aged 60 and over who were tested for COVID-19, 68,556 tested negative and 1,817 tested positive (Figure 1).

We compared the characteristics of the older patients in relation to SARS-CoV-2 infection and COVID-19 deaths using the chi-square test. Patient characteristics, including sex, age, residence, health insurance premium, disability, and the Charlson Comorbidity Index (CCI), were considered, along with whether they had been admitted to a long-term care hospital. Patients were categorized by age in decade groupings, and residence was classified as Seoul, Daegu, Gyeonggi-do, Gyeongsangbuk-do, and others, focusing on areas where COVID-19 cases were frequently observed. In Korea, health insurance premiums are paid in proportion to income level; therefore, health insurance premiums were used as a variable reflecting economic level. Medical aid recipients belong to the low-income class who do not pay health insurance premiums, and those who pay health insurance premiums are classified as first quintile (lowest), second quintile, third quintile, fourth quintile, and fifth quintile (highest), depending on the amount. Disability was defined by whether the person was registered as disabled, and CCI was assigned an updated weight proposed by Quan based on medical records in 2019 (12). In addition, patients admitted to a long-term care hospital were defined as those with a confirmed history of hospitalization within 30 days of the COVID-19 testing date.

In this study, logistic regression analysis was performed to determine the effects of SARS-CoV-2 infection on COVID-19 death in individuals over 60 years of age. The primary outcome was SARS-CoV-2 infection, and the odds ratio (OR) was calculated using logistic regression analysis to analyze the risk of SARS-CoV-2 infection according to admission to a long-term care hospital. In addition, a multivariate logistic regression model was set up to correct the ORs by using sex, age, region of residence, health insurance premium, disability, and CCI score together as independent variables.

The secondary outcome was COVID-19 death, and the OR for the mortality risk was calculated using the same independent variables as those utilized in the SARS-CoV-2 infection model. SAS 9.4 was employed for statistical analysis, and statistical significance was determined based on a value of *p* < 0.05.

### 2.2. Ethics statement

This study conformed to the Korean Guidelines for De-identification of Personal Data and was approved by the Institutional Review Board of Kyung Hee University [IRB No. KHSIRB-21-178(EA)] as a review exemption study. Since this study used de-identified data, the requirement for informed consent was waived by the board.

## 3. Results

An independence (chi-square) test was applied to the test and treatment results, based on the characteristics of the persons tested for COVID-19 and the characteristics of patients with SARS-CoV-2 infection, respectively (Table 1). Sex, age, region of residence, health insurance premium level, disability, CCI score, and admission to a long-term care hospital were confirmed to be related to SARS-CoV-2 infection. In the independence test for the characteristics and death of those infected by SARS-CoV-2, all factors, except the health insurance premium level, were found to be related to treatment results.

**Table 1:** Patients’ baseline characteristics.

	People who tested for COVID-19 (*N* = 70,373)					COVID-19 patients (*N* = 1,817)				
	Non-COVID-19 patients (*n* = 68,556)		COVID-19 patients (*n* = 1,817)		*p* value	Survival (*n* = 1,689)		Death (*n* = 128)		*p* value
	*n*	%	*n*	%		*n*	%	*n*	%	
Sex										
Male	34,495	50.3	726	40.0	<0.001	655	38.8	71	55.5	<0.001
Female	34,061	49.7	1,091	60.0		1,034	61.2	57	44.5	
Age (years)										
60–69	25,794	37.6	1,006	55.4	<0.001	978	57.9	28	21.9	<0.001
70–79	21,936	32.0	493	27.1		467	27.6	26	20.3	
80+	20,826	30.4	318	17.5		244	14.4	74	57.8	
Region of residence										
Seoul	15,371	22.4	43	2.4	<0.001	42	2.5	1	0.8	0.013
Daegu	6,101	8.9	1,311	72.2		1,231	72.9	80	62.5	
Gyeonggi-do	15,611	22.8	57	3.1		54	3.2	3	2.3	
Gyeongsangbuk-do	5,689	8.3	306	16.8		274	16.2	32	25.0	
Others	25,784	37.6	100	5.5		88	5.2	12	9.4	
Health insurance premium										
Medical aid	7,757	11.3	280	15.4	<0.001	257	15.2	23	18.0	0.649
First quintile (lowest)	10,226	14.9	332	18.3		306	18.1	26	20.3	
Second quintile	7,303	10.7	197	10.8		186	11.0	11	8.6	
Third quintile	9,156	13.4	252	13.9		239	14.2	13	10.2	
Fourth quintile	12,473	18.2	315	17.3		290	17.2	25	19.5	
Fifth quintile (highest)	21,641	31.6	441	24.3		411	24.3	30	23.4	
Disability										
No	52,357	76.4	1,489	81.9	<0.001	1,398	82.8	91	71.1	0.001
Yes	16,199	23.6	328	18.1		291	17.2	37	28.9	
CCI score										
0	23,790	34.7	1,047	57.6	<0.001	1,008	59.7	39	30.5	<0.001
1	12,762	18.6	280	15.4		265	15.7	15	11.7	
2	17,214	25.1	334	18.4		283	16.8	51	39.8	
3+	14,790	21.6	156	8.6		133	7.9	23	18.0	
Admission to a long-term care hospital										
No	65,860	96.1	1,698	93.5	<0.001	1,610	95.3	88	68.8	<0.001
Yes	2,696	3.9	119	6.5		79	4.7	40	31.3	

CCI, charlson comorbidity index.

Logistic regression analysis revealed that older patients admitted to long-term care hospitals had a high risk of SARS-CoV-2 infection and COVID-19 death (Table 2). The results of the logistic regression analysis for SARS-CoV-2 infection in 70,373 individuals tested for COVID-19 aged 60 or older, considering sex, age, residential area, health insurance premium level, disability, and CCI, including long-term care hospital admission, were analyzed to determine the effect of SARS-CoV-2 infection. The SARS-CoV-2 infection rate was high in women (OR: 1.42, 95% CI: 1.28–1.58); however, the risk of infection decreased with age. Daegu (OR:51.03, 95% CI:41.52–62.72) and Gyeongsangbuk-do (OR:14.74, 95% CI:11.73–18.52), where cluster infections of COVID-19 started, showed higher infection rates than other regions; Seoul and Gyeonggi-do, the metropolitan areas of Korea, exhibited no statistical difference from other regions. The higher the level of health insurance premiums, the lower the SARS-CoV-2 infection rate. However, there was no difference in the infection rate between the recipients of medical aid and the first quintile of health insurance premiums. Older people with disabilities (OR: 0.81, 95% CI: 0.71–0.93) had a low SARS-CoV-2 infection rate; the higher the CCI score, the lower the infection rate. We found that the main variable in this study, namely hospitalization in a long-term care hospital, had a statistically significant effect on SARS-CoV-2 infection, and the risk of infection among older adults hospitalized in a long-term care hospital (OR:2.91, 95% CI:2.33–3.58) appeared to be high.

**Table 2:** Odds ratios for risk of SARS-CoV-2 infection and COVID-19 death.

	OR for risk of COVID-19 infection (*N* = 70,373)			OR for risk of COVID-19 death (*N* = 1,817)		
	Adjusted OR	95% CI	*p* value	Adjusted OR	95% CI	*p* value
Sex						
Male	1(ref)	1(ref)	1(ref)	1(ref)	1(ref)	1(ref)
Female	1.42	(1.28–1.58)	<·001	0.36	(0.24–0.55)	<0.001
Age (years)						
60–69	1(ref)	1(ref)	1(ref)	1(ref)	1(ref)	1(ref)
70–79	0.73	(0.65–0.83)	<0.001	1.74	(0.99–3.07)	0·054
80+	0.54	(0.47–0.63)	<0.001	6.97	(4.10–11.84)	<0.001
Region of residence						
Others	1(ref)	1(ref)	1(ref)	1(ref)	1(ref)	1(ref)
Seoul	0.70	(0.49–1.00)	0.052	0.28	(0.03–2.36)	0.241
Daegu	51.03	(41.52–62.72)	<0.001	0.52	(0.25–1.05)	0.067
Gyeonggi-do	0.92	(0.66–1.27)	0.601	0.47	(0.12–1.86)	0.283
Gyeongsangbuk-do	14.74	(11.73–18.52)	<0.001	0.70	(0.32–1.53)	0.372
Health insurance premium						
Medical aid	1(ref)	1(ref)	1(ref)	1(ref)	1(ref)	1(ref)
First quintile (lowest)	0.84	(0.70–1.01)	0.064	1.02	(0.53–1.98)	0.944
Second quintile	0.79	(0.64–0.97)	0.024	0.97	(0.43–2.21)	0.941
Third quintile	0.71	(0.59–0.86)	<0.001	0.93	(0.43–2.04)	0.862
Fourth quintile	0.70	(0.59–0.84)	<0.001	1.10	(0.56–2.15)	0.779
Fifth quintile (highest)	0.59	(0.50–0.70)	<0.001	0.68	(0.36–1.30)	0.243
Disability						
No	1(ref)	1(ref)	1(ref)	1(ref)	1(ref)	1(ref)
Yes	0.81	(0.71–0.93)	0.002	1.17	(0.74–1.85)	0.510
CCI score						
0	1(ref)	1(ref)	1(ref)	1(ref)	1(ref)	1(ref)
1	0.58	(0.51–0.68)	<0.001	1.25	(0.66–2.38)	0.488
2	0.45	(0.40–0.52)	<0.001	2.04	(1.23–3.39)	0.006
3+	0.30	(0.25–0.36)	<0.001	1.70	(0.91–3.16)	0.095
Admission to a convalescent hospital						
No	1(ref)	1(ref)	1(ref)	1(ref)	1(ref)	1(ref)
Yes	2.91	(2.33–3.64)	<0.001	3.58	(2.13–6.02)	<0.001

OR, odds ratio; CI, confidence interval; CCI, charlson comorbidity index.

Logistic regression analysis of death risk factors for the 1,817 confirmed COVID-19 patients aged 60 or older revealed that sex, age, and CCI score, including long-term care hospital admission, affected COVID-19 death. Contrary to the risk of SARS-CoV-2 infection, COVID-19 mortality rates in women (OR: 0.36; 95% CI: 0.24–0.55) were high, and the mortality risk increased with age. The residential area, health insurance premium level, and disability in patients with COVID-19 did not have a statistically significant correlation with COVID-19 mortality. However, a statistical difference was confirmed among older adult COVID-19 patients with a CCI score of 0 and 2; unlike infection risk, the mortality risk was higher for confirmed patients with a score of 2 (OR: 2.04, 95% CI: 1.23–3.39). In this study, it was found that hospitalization in long-term care hospitals had a statistically significant effect on COVID-19 death, and the death risk (OR: 3.58, 95% CI: 2.13–6.02) for older patients with COVID-19 hospitalized in long-term care hospitals was also very high.

## 4. Discussion

Logistic regression analysis was applied to SARS-CoV-2 infection and COVID-19 death risk in patients aged 60 or older who were tested for COVID-19. The analysis confirmed that older patients admitted to long-term care hospitals had a higher risk of SARS-CoV-2 infection and death when infected. Long-term care hospital admission was the most important factor for death from COVID-19, excluding age (OR: 3.58; 95% CI: 2.13–6.02). In the analysis of SARS-CoV-2 infection risk factors of individuals tested for COVID-19 aged 60 or older, it was found that sex, age, residential region, health insurance premium level, disability, and CCI score had an effect, as did hospital admission. Further, the analysis of the COVID-19 death risk confirmed that, in persons aged 60 or older, sex, age, and CCI score, along with whether they were admitted to a long-term care hospital, affected their COVID-19 mortality risk.

Indeed, the analysis which examined the risk of SARS-CoV-2 infection and COVID-19 death among older adults aged 60 or older revealed that the rate of SARS-CoV-2 infection was higher in women, and the mortality risk from COVID-19 was higher in men. According to a press release from the Korea Centers for Disease Control and Prevention, the gender distribution of cumulative COVID-19 confirmed cases until May 30, 2020 was 41.8% males and 58.2% females; during this period, when COVID-19 was in the early stages, many infections were reported in women (13). The difference in the risk of SARS-CoV-2 infection by sex in this study can be attributed to the difference in the sex distribution of infected people during the early stages of COVID-19. Gender differences in COVID-19 death risk have been confirmed in many previous studies; furthermore, it is known that mortality is particularly high in older adult males (14–16). Differences in the clinical outcome of infectious diseases by sex occur in all age groups; males have a higher disease burden, and the same appears in SARS-CoV-2 infection (17–20). In a study by Yanez et al. (14), analysis of COVID-19 data reported by 16 countries confirmed that COVID-19 deaths were highly correlated with age and sex, and that male mortality rates were 77% higher than in females. A study by Peckham et al. (15) confirmed that men had a high risk of COVID-19 death and admission to intensive care units; this was determined through a meta-analysis based on COVID-19 data reported worldwide. The above-mentioned authors also noted that these results stem from fundamental differences in gender immune responses (15). Moreover, a study by Scully et al. (16) introduced sex features of immune responses and explained the causes of high COVID-19 severity in men.

In the current study, where the data analyzed came from older adults, it was confirmed that the higher the age, the lower the risk of SARS-CoV-2 infection, and the higher the mortality risk from COVID-19. During the early stages of the COVID-19 pandemic, relatively active groups were more likely to be infected, and this seemed to be related to the lower likelihood of COVID-19 infection as age increased (21). However, it seems that older patients have a high mortality risk, since their immunity diminishes as they age (22); furthermore, previous studies have reported that older patients are at a higher risk of COVID-19 death (14, 23, 24).

Differences were observed in SARS-CoV-2 infection rates among older adults according to residential region, health insurance premium level, and disability status. Daegu and Gyeongsangbuk-do, where cluster infections occurred in the early days of the COVID-19 pandemic, had high rates of SARS-CoV-2 infection. The results indicated that, the higher the health insurance premium level, the lower the SARS-CoV-2 infection rate, which means that the risk of SARS-CoV-2 infection was lower in the group with a higher economic level. These results suggest that people with low economic levels are more likely to be infected with SARS-CoV-2 because they are exposed to environments wherein it is difficult to comply with social distancing (25–27). Additionally, older adults without disabilities had a higher SARS-CoV-2 infection rate; it may be assumed that the risk of infection was higher because they engaged in more outdoor activities than did older adults with disabilities. A previous study in the United States found that counties with a higher proportion of disabled people had a lower SARS-CoV-2 infection rate, attributed to lower levels of mobility (21).

Despite the differences in SARS-CoV-2 infection rates according to residential area, health insurance premium level, and disability among older adults aged 60 years or older, no difference in mortality was found. In other words, there are statistical differences in SARS-CoV-2 infection rates according to the factors that cause social inequality, but there is no difference in COVID-19 mortality. Contrastingly, a study by Kim et al. (28) confirmed that social vulnerability was significantly related to an increase in COVID-19 mortality using Chicago COVID-19 mortality data. In addition, Patel et al. argued that COVID-19 mortality was higher among people with lower socioeconomic status because they are exposed to environments that are more vulnerable in terms of healthcare (e.g., low access to medical care) (29). The difference between the results of this study and those of existing studies suggests that Korea’s health insurance system is realizing Universal Health Coverage. The Korean government has supported medical expenses for COVID-19 testing, hospitalization, and home treatment prior to July 11, 2022, and these policies may be related to no difference in mortality due to social factors (30). This means that COVID-19 mortality rates according to socioeconomic factors can be improved with appropriate national policy interventions.

As the CCI of the older adults increased, the SARS-CoV-2 infection rate decreased. The association between CCI and SARS-CoV-2 infection in older adults can be explained by lower levels of mobility. As older adults with disabilities have a lower risk of infection, it can be understood that the people with poor health levels are less likely to be exposed to the risk of SARS-CoV-2 infection due to low outdoor activity (21). In contrast, older patients with COVID-19 exhibited a lower mortality rate in the absence of comorbidities, and many previous studies have confirmed that comorbidities increase the mortality risk from COVID-19 (31–34).

Older patients admitted to long-term care hospitals were found to have a very high risk of SARS-CoV-2 infection and death, even after correcting for sex, age, and severity. In particular, it was confirmed that admission to long-term care hospitals was a very important risk factor for death. Previous studies have affirmed a high risk of SARS-CoV-2 infection among residents, staff, and visitors of long-term care facilities (35, 36). This suggests that long-term care facility environments, such as care environments with high physical contact density between residents and workers and use of common spaces, are vulnerable to the spread of infectious diseases (37). In addition, long-term care facility residents are at high risk of COVID-19 death. In a study by Fisman et al. (9), the mortality risk from COVID-19 was 13 times higher among older adults at long-term care facilities than in those living in the community. Sepulveda et al. (38) compared COVID-19 mortality in long-term care facility residents in 12 OECD countries and noted high mortality owing to the characteristics of older residents, such as immune aging and high prevalence of chronic diseases. This study differs from previous works in that the risk of SARS-CoV-2 infection and COVID-19 death in older patients admitted to a long-term hospital was high, despite the fact that the effects of chronic diseases were corrected through CCI.

The limitation of this study is that chronic disease and severity (CCI) were defined based on the ICD-10 code of the health insurance claim data. Due to the nature of administrative data, there are concerns about under- or over-coding diagnoses (39). To reduce the limitations of these administrative data, the CCI calculation method was applied conservatively only for the main diagnosis at the time of admission, although limitations remain. Nonetheless, it is meaningful to derive generalizable results by analyzing all older people who have been tested for COVID-19 using Korea’s national data.

This study confirmed that older patients admitted to long-term care hospitals were vulnerable to SARS-CoV-2 infection and had a high mortality risk. Although Korean long-term care hospitals are medical institutions, their characteristics, which are similar to those of long-term care facilities, allow infectious diseases to spread rapidly. Additionally, they are exposed to a high mortality risk from SARS-CoV-2 infection. Active infection prevention measures are important when it comes to preventing the spread of SARS-CoV-2 infection and death among patients admitted to long-term care hospitals, the latter of whom are at a high risk of infection through contact with workers such as medical staff and caregivers (40). Therefore, it is necessary to strengthen quarantine policies and increase the effectiveness of detecting and mitigating the spread of COVID-19 via, for example, infectious disease management monitoring systems for workers. The results of this study can contribute to policy decisions regarding the management of infectious diseases in long-term care hospitals. Meanwhile, the current study confirmed differences according to socioeconomic status in the case of COVID-19 infection among older adults, although no difference in mortality was observed. This suggests that Korea’s policy of providing COVID-19-related testing and treatment free of charge was effective. In health emergencies, the government’s policy of compensating for all medical expenses can be effective in improving clinical outcomes and can be applied in similar situations in the future.

## Data availability statement

The data analyzed in this study is subject to the following licenses/restrictions: The datasets generated and/or analyzed in the current study are available from the corresponding author upon reasonable request. Requests to access these datasets should be directed to National Health Insurance Sharing Service, https://nhiss.nhis.or.kr/bd/ab/bdaba000eng.do.

## Author contributions

I-HO conceived and designed the study. J-YS and SK designed the study and performed the analyses. J-YS, SK, and ML interpreted the data and study findings. J-YS, SK, ML, and I-HO are responsible for writing and editing the manuscript. All authors contributed to the article and approved the submitted version.

## Conflict of interest

The authors declare that this research was conducted in the absence of any commercial or financial relationships that could be construed as a potential conflict of interest.

## Publisher’s note

All claims expressed in this article are solely those of the authors and do not necessarily represent those of their affiliated organizations, or those of the publisher, the editors and the reviewers. Any product that may be evaluated in this article, or claim that may be made by its manufacturer, is not guaranteed or endorsed by the publisher.
