# Supplementary material for: Increased risk of SARS-CoV-2 infection and COVID-19 death among older patients at long-term care hospitals in Korea
Source: Front Public Health. 2023 Jul 25;11:1235745. doi: 10.3389/fpubh.2023.1235745 (PMC10407124; doi:10.3389/fpubh.2023.1235745)
Supplement: Supplementary file 1 [file Table_1.DOCX]

Supplementary Material

Increased risk of SARS-CoV-2 infection and COVID-19 death among older patients at long-term care hospitals in Korea

Jeong-Yeon Seon, Sunjea Kim, Min Kyoung Lim, In-Hwan Oh^*^

*** Correspondence:** In-Hwan Oh: parenchyme@gmail.com

# Supplementary Data

**Supplement 1. Subjects for inspection according to the revised COVID-19 Response Guidelines**

| **Revision**  **(Publication date)** | **Who should be tested for COVID-19** |
| --- | --- |
| **1st (2020.01.04.)** | ·A person who develops a fever or severe respiratory symptoms (e.g., pneumonia) within 14 days of visiting the Huanan seafood market  ·A person who develops a fever or respiratory symptoms (e.g., cough, shortness of breath, etc.) within 14 days of visiting the Huanan seafood market  ·A person who develops a fever or severe respiratory symptoms (e.g., pneumonia) within 14 days of visiting Wuhan |
| **2nd (2020.01.08.)** | ·A person who develops pneumonia or symptoms of suspected pneumonia (e.g., shortness of breath with fever) within 14 days of visiting the Huanan seafood market  ·A person who develops a fever or respiratory symptoms (e.g., cough etc.) within 14 days of visiting the Huanan seafood market  ·A person who develops pneumonia or symptoms of suspected pneumonia (e.g., shortness of breath with fever) within 14 days of visiting Wuhan |
| **3rd (2020.01.17.)** | ·A person who develops pneumonia or symptoms of suspected pneumonia (e.g., shortness of breath with fever) within 14 days of visiting Wuhan  ·A person who develops the following symptoms within 14 days of contact with a confirmed case during the confirmed case’s symptomatic period:  - a fever or respiratory symptoms (e.g., cough etc.), pneumonia or symptoms of suspected pneumonia (e.g., shortness of breath with fever)  ·A person who develops a fever or respiratory symptoms (e.g., cough etc.) within 14 days of visiting Wuhan |
| **4th (2020.01.27.)** | ·A person who develops a fever or respiratory symptoms (e.g., cough, sore throat, etc.) within 14 days of visiting Hubei province  ·A person who develops pneumonia within 14 days of visiting Mainland China |
| **5th (2020.02.06.)** | ·A person who develops a fever or respiratory symptoms (e.g., cough, sore throat, etc.) within 14 days of visiting Mainland China  ·A person who develops a fever or respiratory symptoms (e.g., cough, sore throat, etc.) within 14 days of contact with a confirmed case during the confirmed case’s symptomatic period  ·A person suspected of having COVID-19 according to a physician’s judgment |
| **6th (2020.02.19.)** | ·A person who develops a fever or respiratory symptoms (e.g., cough, sore throat, etc.) within 14 days of visiting Mainland China (including Hong Kong and Macau)  ·A person who has unknown pneumonia that requires hospitalization according to a physician’s judgment  ·A person who develops a fever or respiratory symptoms (e.g., cough, etc.) within 14 days of visiting countries, territories, or areas with reported COVID-19 cases  ·A person suspected of having COVID-19 according to a physician’s judgement |
| **7th (2020.03.02.)** | ·A person who develops symptoms within 14 days of contact with a confirmed case during the confirmed case’s symptomatic period  ·A person suspected of having COVID-19 (unknown pneumonia etc.) according to a physician’s judgement  ·A person who develops a fever or respiratory symptoms (e.g., coughing, shortness of breath, etc.) within 14 days of visiting countries with local COVID-19 transmissions  ·A person who develops a fever or respiratory symptoms (e.g., coughing, shortness of breath, etc.) within 14 days and is epidemiologically related to domestic COVID-19 outbreaks |
| **8th (2020.05.11.)** | ·A person who develops symptoms within 14 days of contact with a confirmed case during the confirmed case’s symptomatic period  ·Main symptoms: fever, cough, shortness of breath, chills, myalgia, headache, sore throat, loss of taste/smell sense, or pneumonia etc.  ·A person suspected of having COVID-19 according to a physician’s judgement  ·A person who develops symptoms within 14 days of overseas travel  ·A person who develops symptoms within 14 days and is epidemiologically related to a domestic COVID-19 outbreak |
